# Perspectives acquired through long-term epidemiological studies on the Great East Japan Earthquake

**DOI:** 10.1186/s12199-017-0615-x

**Published:** 2017-03-15

**Authors:** Toru Tsuboya, Mariko Inoue, Michihiro Satoh, Kei Asayama

**Affiliations:** 1 Liaison Center for Innovative Dentistry / Department of International and Community Oral Health, Tohoku University Graduate School of Dentistry, 4-1, Seiryo-cho, Aoba-ku, Sendai, 980-0872 Japan; 2 Graduate School of Public Health, Teikyo University, 2-11-1 Kaga, Itabashi-ku, Tokyo, 173-8605 Japan; 3 Division of Public Health, Hygiene and Epidemiology, Faculty of Medicine, Tohoku Medical and Pharmaceutical University, 4-4-1 Komatsushima, Aobaku, Sendai, 981-8558 Japan; 4 Department of Hygiene and Public Health, Teikyo University School of Medicine, Tokyo, Japan; 5 Department of Planning for Drug Development and Clinical Evaluation, Tohoku University Graduate School of Pharmaceutical Sciences, 2-1 Seiryo-cho, Aoba-ku, Sendai, 980-8575 Japan

**Keywords:** Great East Japan Earthquake, Tsunami, Cohort study, Resilience, Disaster

## Abstract

The Great East Japan Earthquake (GEJE) and subsequent tsunamis that occurred in 2011 caused extensive and severe structural damage and interrupted numerous research activities; however, the majority of such activities have been revived, and further public health researches and activities have started to follow the population affected by the disaster. In this mini-review, we overview our recent activities regarding epidemiologic studies in Miyagi Prefecture, the region most affected by the GEJE. Through our study processes, we were able to identify the particular characteristics of vulnerable populations, and provide ideas that may help save lives and reduce the amount of damage caused by a future disaster. Long-term follow-up and care of survivors is essential in affected areas, and health professionals should pay particular attention to various diseases, e.g., cardiovascular complications and mental disorders. Furthermore, building up resilience and social relationships in the community is beneficial to survivors. Ongoing cohort studies conducted before disasters can help minimize biases regarding the survivors’ pre-disaster information, and emerging cohort studies after disasters can find potential helpful novel indices. To identify characteristics of vulnerable populations, save lives, and reduce the amount of damage caused by a future disaster, constant research that is consistently improved by new data needs to be performed.

## Introduction

The Great East Japan Earthquake (GEJE) and subsequent tsunamis occurred at 14:46 on March 11, 2011 [1, 2]. The GEJE had a magnitude of 9.0 on the Richter scale, making it the most powerful earthquake in Japan and the fourth in the world since modern record-keeping began in 1900. The GEJE triggered a series of devastating tsunamis, that caused extensive and severe structural damage and interrupted numerous research activities, such as ongoing community-based cohort and epidemiological studies. However, the majority of these research activities have since been revived, and further public health research and activities have started to follow the population most affected by the GEJE. Based on the recent seismological findings, a simple ‘characteristic earthquake’ model for the long-term forecast is difficult to apply, e.g., recurrence of Nankai Trough Earthquake with an interval of ≈ 100 years has been identified but the sizes of recurrent earthquakes vary according to tsunami deposits [3]. We should therefore improve managing disaster risks for a resilient future (https://www.gfdrr.org/sites/gfdrr/files/sendai/sendai.html (2016.10.17)). In this mini-review, we provide a brief overview of our recent activities based on epidemiological studies conducting in Miyagi Prefecture , the region most affected by the disaster, and discuss what we need for saving lives and reducing damage after a disaster. We further aim to specify a significance of consistent improvement of longitudinal studies.

## Iwanuma project — social cohesion and mental health

The Iwanuma Project [4, 5] is a part of the general population-based Japan Gerontological Evaluation Study (JAGES) [6, 7]. The JAGES was a nationwide study established in 2010 to prospectively examine the determinants of healthy aging [6]. Approximately 48% of Iwanuma City, which is located in a southern coastal area in Miyagi Prefecture, was inundated by the tsunamis after the GEJE (Table 1). Approximately 2.5 years after the disaster, we carried out a follow-up survey among 4,380 survivors in Iwanuma aged ≥65 years old [4, 5].

**Table 1:** Population sampling methods and characteristics of each study

	Iwanuma Project^a^ [4, 5]	Health and Life Revival Council in Ishinomaki District (RCI) [13, 14, 16–18]	Tohoku Study of Child Development (TSCD) [2, 19–23]
Place of municipality	Iwanuma	Ishinomaki	Sendai and the Sanriku coastal areas of Miyagi Prefecture
Population of the municipality in Sep 2015	44,149	148,968	>1 million
Damage from GEJE and subsequent tsunamis on Mar 2011	187 died, 48% of the land was inundated.	3,055 died, 420 missing, 270 certified as disaster-related death.	On TSCD, 2 mothers and 3 children died, 10 families missing, the investigation was suspended for 5 months.
Sampling frame	8,576 residents ≥65 years	≈8,700 survivor families living only on the 2nd floor of their damaged houses	Neonates born at 36–42 weeks of gestation with a birth weight of ≥2400 g.
Recruitment period	Aug 2010	Oct 2011 to Mar 2012 as 1st-term survey	Jan 2001 to Sep 2005
Latest follow-up period	Oct 2013 to Jan 2014	Apr 2013 to Jan 2014 as 2nd-term survey	Mar 2013 as the 7-year-old survey
Number in each cohort database	4,380 survivors	4,176 households (1st-term); 4,023 (2nd-term)	1,348 mother-offspring pairs from 3 maternity hospitals
Follow-up rate	82.1%	n/a (2 surveys not yet fully linked)	70.7%
Outcome reported	PTSD symptomatology, mental health, physical function, social activies	Sleep problems, psychological distress, symptoms, access to health services	Home and conventional blood pressure, verbal, performance, and full-scale IQ, autonomic nervous indicators

*GEJE* the Great East Japan Earthquake, *PTSD* posttraumatic stress disorder, *IQ* intelligence quotient

^a^Iwanuma Project is a part of the Japan Gerontological Evaluation Study

Among the analytic sample of the Iwanuma residents, 38.0% reported losing loved ones such as friends or relatives, 59.0% reported damage to their property resulting from the GEJE, and 11.4% reported having symptoms of severe posttraumatic stress disorder, [4] which was measured using the Screening Questionnaire for Disaster-Related Mental Health [8]. Based on the spatial Durbin model, which can capture the spatial spillover association of community social cohesion, [9] social cohesion was significantly associated with a risk of posttraumatic stress disorder symptoms at both the individual-level (odds ratio [OR], 0.87; 95% confidence interval [CI], 0.77–0.98) and community-level (OR, 0.75; 95% CI, 0.63–0.90) [4]. Loss of loved ones and damaged housing at the individual level and an interruption in access to internal medicine and psychiatry services were also associated with a higher risk of posttraumatic stress disorder symptoms (*p* < 0.01) [4]. We would emphasize that our findings were not affected by recall bias because we obtained information on Iwanuma residents both before and after the GEJE. Social cohesion among neighbors may help to explain the community-level variations in the occurrence of posttraumatic stress disorder after natural disasters [4].

We further found associations between experiences of disaster-related damage and changes in depressive symptoms among survivors, [5] based on the dichotomous 15-items Geriatric Depression Scale (GDS), which has been validated in both English [10] and Japanese [6]. We evaluated personal experiences of damage caused by the GEJE and tsunamis based on previous studies on mental health among survivors after natural disasters, in consideration of the local culture [11, 12]. Among 3,464 eligible participants with complete data, the mean (SD) GDS scores at baseline and follow-up were 3.74 (3.5) and 3.84 (3.4), respectively. The changes in GDS scores between the two surveys were more pronounced among men than among women (0.17 *versus* 0.05). Multivariate-adjusted linear regression analysis showed that experiences of property loss were significantly associated with worsening GDS scores: the non-standardized coefficient of the model was 1.40 (95% CI, 0.96–1.83) for total housing loss and 0.47 (95% CI, 0.21–0.74) for loss of an automobile. The impact on loss of family members was also significant, whereas the effect size of the GDS was relatively small, at 0.23 (95% CI, 0.02–0.44). Both loss of employment (*n* = 186, 5.1%) and disruption of access to psychiatric care (*n* = 17, 0.5%) were also associated with worsening GDS scores (*p* < 0.0001). In a community-dwelling elderly population, loss of employment, disruption of access to psychiatric care, and personal experiences of property damage predict a worsening of depressive symptoms, and have a more pronounced and lingering impact compared with experiencing the loss of a loved one [5].

## Health and life revival council in Ishinomaki District — community support

Ishinomaki is one of the municipalities most severely damaged from the GEJE and subsequent tsunamis (Table 1) [13, 14]. After the GEJE, approximately 8,700 families in Ishinomaki remained at home, despite serious structural damage; many of these residents were living entirely on the second floor of their homes because the tsunami had completely swept away everything on the first floor [13, 14]. This vulnerable population, referred to as the stay-at-home victims, was provided with less support by governmental agencies than those living in temporary shelters. To identify and evaluate stay-at-home survivors for providing more rapid and appropriate support in response to their needs, the Health and Life Revival Council in Ishinomaki District (RCI) conducted a face-to-face survey. We used a semi-structured questionnaire composed of the following three sections: household demographics; social background; and health condition of individuals most in need of medical support. The first-term survey was started approximately 6 to 12 months after the GEDE (Table 1) [14].

Initially, data from 4,176 households were analyzed, with 4,023 household members agreeing to be interviewed using the written form in the first-term and future surveys [14]. Factors associated with sleep difficulties which frequently begin as stress-related phenomena including natural disaster [15] were loss of pleasure in life (OR, 1.37; 95% CI, 1.07–1.76), living on a pension (versus living on salary: OR, 1.58; 95% CI, 1.09–2.27) or public livelihood assistance (versus living on salary: OR, 4.40; 95% CI, 1.53–12.65), or women (OR, 2.67; 95% CI, 2.07–3.46) [14]. Interacting with neighbors by visiting one another was positively associated with sleep (OR, 0.42; 95% CI, 0.24–0.74) [14]. Among survivors living at home around 2 years after the GEJE, participants with emotional social support (OR, 2.05; 95% CI, 1.35–3.11) and informational social support (OR, 1.55; 95% CI, 1.08–2.21) had lower rates of sleep difficulties, [16] suggesting that relatively strong neighborhood networks help prevent sleep difficulties. Loss of pleasure in life (OR, 1.25; 95% CI, 1.00–1.57), changes in family structure (OR, 1.46; 95% CI, 1.15–1.85), and changes in work status (OR, 1.30; 95% CI, 1.06–1.59) were also significant factors associated with psychological distress [17]. Contrary to the hypothesis, the elderly (>65 years) were more likely to have close neighbors and social/family interactions; non-elderly men living alone represented the highest proportion of people without social/family interactions, and persons living alone were less likely to have social/family interactions [18]. Strengthening social ties before and after disasters is important in building a resilient community capable of supporting the physical and mental health of its citizens.

## The Tohoku study of child development — comprehensive development of children

The Tohoku Study of Child Development (TSCD) is a prospective birth cohort study primarily carried out to investigate the potential effects of perinatal exposure to environmental pollutants on the neurobehavioral development of children (Table 1) [19, 20]. After conducting periodic surveys, self-measured home blood pressure of children at 7 years of age was recorded; however, the GEJE caused a disruption in the data collection among those in the affected rural coastal areas [2, 21]. Although several participants were victimized (Table 1), [2] further research equipment, such as home blood pressure devices, were supplied relatively quickly, and data were recovered from a backup system as well as from the internal storage of each device, and the examinations were successfully resumed 5 months after the GEJE [2].

Maternal gestational hypertension did not affect home blood pressure in offspring, but it did strongly affect maternal home blood pressure, even 7 years after giving birth [22]. Home blood pressure among children with 7 years of age who received long-term (mean, 11.3 months) breastfeeding as a major source of nutrition was significantly lower than that among children who received short-term (mean, 5.1 months) breastfeeding (92.9/55.1 *versus* 94.7/56.4 mm Hg systolic/diastolic blood pressure, respectively; *p* = 0.006/0.04), suggesting that long-term breastfeeding had a protective effect against elevated blood pressure even in young children [23]. Furthermore, Tatsuta and colleauges assessed the neurodevelopmental effects of the GEJE [2]. Verbal intelligence quotient (IQ) at 7 years of age was significantly lower in a post-disaster group than in a pre-disaster group after the adjustment of preceding neurodevelopmental scores taken at 32 and 40 month olds (*p* = 0.001). In contrast, no significant differences were found in performance IQ, full-scale IQ, or autonomic nervous indicators (*p* = 0.053). This finding implies the indispensable effect of interrupted schooling [2].

The TSCD offered the opportunity to start a new line of longitudinal research into cardiovascular risk factors, such as blood pressures, as measured by conventional and self-measured at home in children from childhood to adolescence along with their mothers [21]. The TSCD is currently investigating the comprehensive health and development of children, including the long-term multilateral impact of the inevitable effects of the disaster [2].

## Other studies in Miyagi Prefecture — hypertension and diabetes

Our research group has engaged in other collaborative research projects, such as local hospital-based surveys. Satoh and coworkers compared home blood pressure just before and after the GEJE in 142 hypertensive patients (40.8% women; mean age, 68.1 years) [24]. The mean home systolic blood pressure of 126.9 mm Hg just before the GEJE increased to 129.3 mm Hg just after the GEJE. This increase remained significant 2 weeks after (*p* = 0.03) but disappeared by 4 weeks after the GEJE (*p* = 0.2). Among the 142 hypertensive patients, only 10 could measure their home blood pressure in the morning before the GEJE and during the subsequent 3 days after the GEJE. A steep elevation in home blood pressure was observed in those 10 patients, with the difference of 11.6 mm Hg in home systolic blood pressure between the day of and the day after the GEJE (*p* = 0.02) [24].

Tanaka and colleagues investigated the impact of the GEJE on glycemic control based on 497 diabetic patients who had been followed at hospitals in the devastated Tohoku area [25]. Notably, the HbA1c levels of the diabetic patients were not elevated at 1 month after, and were significantly decreased at 3 months after as compared with before the GEJE [25]. This might have been the result of weight loss due to energy restrictions. Meanwhile, impaired endogenous insulin secretory capacity was associated with worsening of glycemic control after the GEJE [26]. The increased blood pressure or worsening of glycemic control might have led to an increase in the risk of cardiovascular disease just after the disaster [27, 28].

However, these studies [24–26] were only based on participants who could measure their blood pressure at home or visit clinics immediately after the GEJE. Survivors after a disaster would be expected to have higher cardiovascular risk because they cannot afford to take care of their health due to the loss of their home or loved ones. However, there are limitations in investigating the precise effects of a disaster on such survivors unless pre-disaster health data are available. For the management of hypertension in disaster conditions, a special network system for supporting continuous self-measurement of home blood pressure has been proposed [28]. Although it is impossible to monitor everyone after a large-scale disaster, daily home blood pressure measurement among survivors would be useful for investigating the effects of a disaster in relation to cardiovascular complications [24, 29].

## Perspectives

We reviewed several studies in which we have taken part in on social, [4–6, 13, 14, 16–18] birth, [2, 19–23] and cardiovascular [21–27] epidemiologies regarding the GEJE. Through these study processes, we could help identify same particular characteristics of vulnerable populations and provide further ideas for saving lives and reducing damage after future disasters, e.g., by building social capital or social networks within communities. To ensure the ability to respond to such a disaster promptly, our findings can also assist in building a model for collaboration between academic institutions and non-governmental as well as governmental organizations. Both long-term follow-up and care of survivors in the affected areas are essential, and health professionals should pay special attention to various diseases such as cardiovascular complications and mental disorders. Besides providing sufficient medical care, building up resilience and social relationships in the community is expected to be beneficial to survivors. Ongoing cohort studies before disasters [2, 4–6, 19–23] can minimize biases regarding pre-disaster information for survivors, whereas emerging cohort studies after disasters [13, 14, 16–18, 24–27] can identify potentially useful novel indices. Constant research that is consistently improved by new data, therefore, needs to be performed.
